# Exploring the biomedical potential of a novel modified glass ionomer cement against the pandrug-resistant oral pathogen *Candida albicans* SYN-01

**DOI:** 10.1080/20002297.2023.2195741

**Published:** 2023-03-30

**Authors:** Nessma A. El Zawawy, Samy El-Safty, El-Refaie Kenawy, Sara Ibrahim Salem, Sameh S. Ali, Yehia A.-G. Mahmoud

**Affiliations:** a Botany Department, Faculty of Science Tanta University, Tanta, Egypt; b Biomaterials Department, Faculty of Dentistry, Tanta University, Tanta, Egypt; c Polymer Research Group, Department of Chemistry, Faculty of Science Tanta University, Tanta, Egypt

**Keywords:** Glass ionomer cement, chitosan derivatives, *Candida albicans*, dental caries, neuraminidase

## Abstract

Dental caries is an infectious disease that is a major concern for dentists. *Streptococci* and *Lactobacilli* were long thought to be the primary etiology responsible for caries. *Candida albicans* with acidogenic and aciduric characteristics has recently been implicated in the onset and progression of cariogenic lesions. Moreover, due to the increased resistance to common antimicrobials, the discovery of innovative candidates is in high demand. Therefore, our study might be the first report that explores the efficacy of glass ionomer cement (GIC) incorporated with a newly modified carboxylated chitosan derivative (CS-MC) against multidrug-resistant (MDR) and/or pandrug resistant (PDR) *C. albicans* isolated from the oral cavity. In this work, four CS-MC-GIC groups with different concentrations were formulated. Group four (CS-MC-GIC-4) gave a significant performance as an anticandidal agent against selected PDR *Candida* strain, with an obvious decrease in its cell viability and high antibiofilm activity. It also, enhanced all the mechanical properties and supports cell viability of Vero cells as a nontoxic compound. Moreover, CS-MC-GIC-4 inhibited neuraminidases completely, which might provide a novel mechanism to prevent dental/oral infections. Thus, findings in this study open up new prospect of the utilization of CS-MC-GIC as a novel dental filling material against oral drug-resistant *Candida*.

**Figure:**
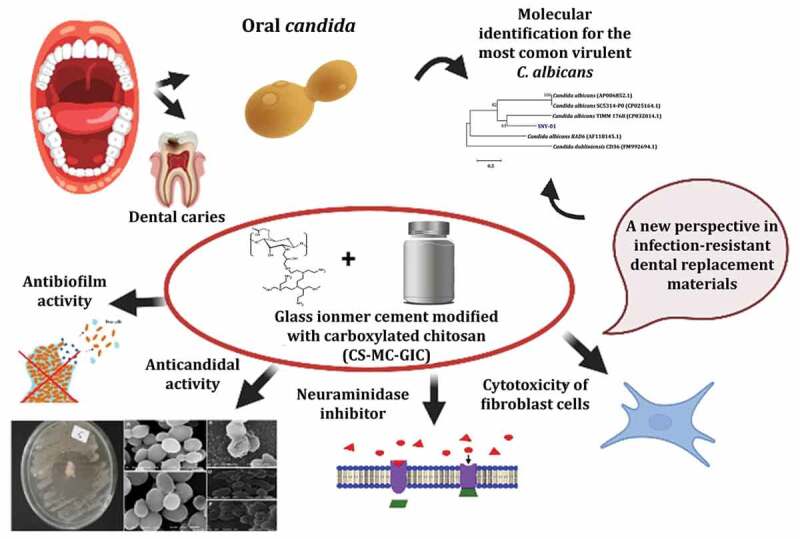

## Introduction

Dental caries is a multifactorial disease caused by a variety of factors, including cariogenic microorganisms, a high carbohydrate intake, poor oral hygiene, malnutrition, and a low socioeconomic status [1,2]. Dental caries is caused by the demineralization of the hard tissues, which leads to the destruction of the organic matter of the tooth, which is frequently caused by acid production by oral microorganisms. The acidic environment may be caused by metabolic end products of dental plaque, which are typically organic acids produced through carbohydrate fermentation [3–6]. *Streptococci* and *Lactobacilli* were thought to be the primary causative agents of human dental caries [7–11]. Numerous studies have recently implicated other oral microbiota, primarily acidogenic and aciduric microorganisms, in dental decay [7,10,12,13]. *Candida* yeast is also included in this newly proposed cariogenic microbiota [8,14–20]. Due to its virulence and pathogenicity, *Candida,* despite being considered commensal, can cause serious opportunistic infections. The presence of *Candida albicans* in the oral cavity should be considered as a risk factor for dental caries [21], as one study found that the oral cavity of children with healthy teeth is almost devoid of *Candida albicans* [22].

*Candida* spp. can colonize several oral cavity surfaces, including the tongue, palate, cheek, and hard surfaces of teeth. As a result of oral surface colonization, *Candida* can present in saliva [8,15,19,23–25]. *Candida* is dimorphic [3] surviving as yeast as well as pseudohyphal or hyphal forms [26]. This characteristic is known as the major virulence determinant. *Candida albicans* also exhibits other pathogenic characteristics, such as interfering with the host’s immune system and producing a variety of metabolites and hydrolyzing enzymes [11]. Moreover, in the last decade, invasive oral candidal infections have progressively grown faster due to the increasing rate of multi-drug and/or pan-drug resistance (MDR/PDR), but also the fact that there are much fewer drug classes available than bacterial infections [27–29]. MDR/PDR to various commercially available antifungal agents has turned into a difficult problem that cannot be solved. Resistance to three or more antifungal classes is referred to MDR. However, resistance to all agents in all antifungal classes is defined as PDR [30]. Indeed, the rapid emergence of antimicrobial resistance traits poses a significant challenge and reduces treatment options globally, necessitating the urgent development of new, broadly effective, and cost-effective therapeutic strategies. Therefore, there is a great need to define the specific role of oral/dental *Candida* species in the etiology of infection and find novel preventive strategies against such resistant strains.

Neuraminidase, or sialidase, is an exoglycosidase hydrolyzing α-linkage of the terminal sialic acids of various sialoglycoconjugates in diverse organisms, including viruses and microorganisms [31]. This enzyme modifies cell-surface-located sialoglycoconjugates, which play important roles in the regulation of cell-to-cell and cell-to-molecule interactions by mediating cell recognition or adhesion processes [32]. Neuraminidases are widely expressed as virulence factors by several mucosal pathogens [33]. Surprisingly, recent evidence suggests that neuraminidase activity may influence biofilm formation [34]. Although neuraminidase production of some microorganisms was considered the main virulence factor to cause infections, the mechanism of fungal neuraminidase in dental caries is not clearly defined [35,36]. Thus, our study may be, up to the moment, the first to provide an insight into the role of fungal neuraminidase enzyme in dental infections.

Glass ionomer cements (GICs) are considered an integral part of the biomaterials that are essential to accomplishing daily dental practices. That is why these materials are frequently investigated in an attempt to improve their properties, particularly their biological and mechanical ones [37,38]. Incorporating a component capable of producing an antimicrobial activity into the GICs has been an important area of research among authors [39,40]. This approach was thought by many scientists to enhance the capability of these materials to withstand the oral microorganisms that may invade the tooth structure. In some investigations, the addition of polymers containing quaternary ammonium salt (QAS) or quaternary phosphonium salt (QPS) was tried. It was reported that these additives have antimicrobial activities and can be useful to enable GICs to overcome secondary caries [41]. Chlorhexidine (CHX), in the form of chlorhexidine acetate, chlorhexidine gluconate, or chlorhexidine hydrochloride, was added to GICs and accepted by many authors because of its antibacterial activities against cariogenic bacteria [42]. Incorporation of CHX into GICs, despite the detrimental effect on physical and mechanical properties and bonding capacity, was said to have a positive impact on fluoride release, which is considered a powerful anti-cariogenic component [43]. Moreover, furanone-modified GICs were also introduced and proved to have long-term antibacterial activities [44]. The addition of propolis to GICs was also studied, and the results revealed an antibacterial activity against different bacteria, particularly [45]. In the same stream, a variety of antibacterial polymers were added to GICs, such as epigallocatechin-3-gallate (EGCG), which can act as a promising antimicrobial agent [46]. New advances in using chitosan derivatives may provide the means to developing such a material [44].

In this study, the development of a GIC modified with carboxylated chitosan (CS-MC) is reported. Several publications have described chitosan as a natural linear amino-polysaccharide that has different biological properties, such as biodegradability, biocompatibility, and non-toxicity [47]. Despite these benefits, chitosan showed poor solubility in water and acidic environments. As a result, chemical modification as graft copolymerization is considered an appealing strategy for modifying the chemical and physical characteristics of chitosan among different techniques of modification to solve challenges of chitosan solubility [48]. Grafting chitosan facilitates the synthesis of functional derivatives by covalently attaching a molecule, the graft, to the backbone of the chitosan, giving different modified chitosan derivatives such as aminated chitosan and carboxylated chitosan, which have a wide range of pharmacological properties [49].

**Figure 1.**
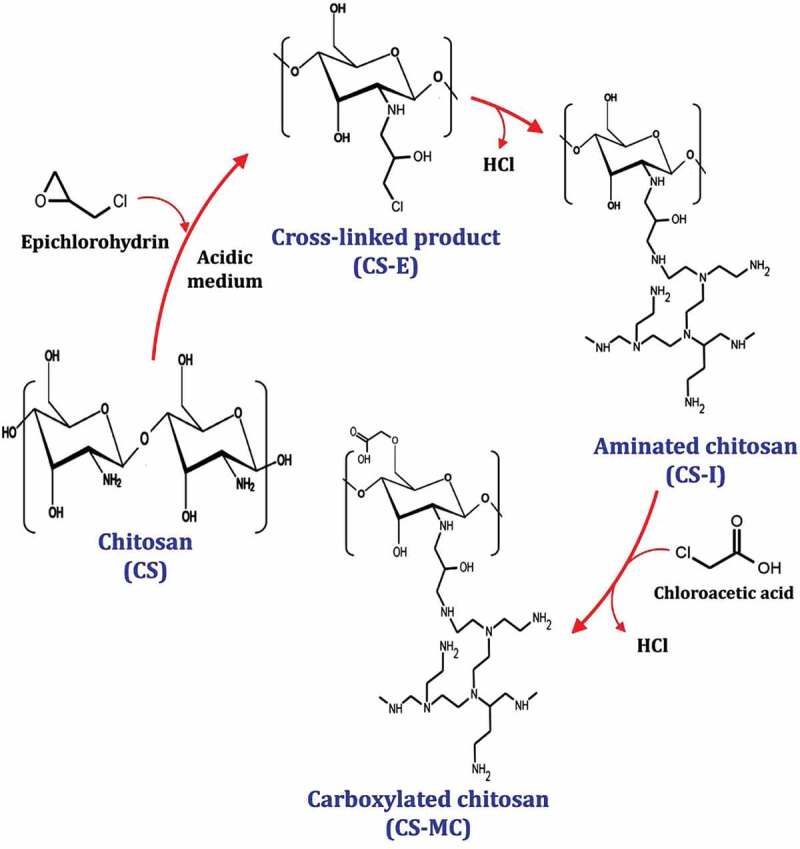
Preparation of carboxylated chitosan based on the graft copolymerization reaction.

To the best of our knowledge, the preparation of a novel modified GIC (CS-MC-GIC) has not been reported previously. Therefore, our group has recently developed a novel carboxylated chitosan (CS-MC) by a graft copolymerization reaction in three steps [50] as shown in Figure 1: (i) Chitosan (CS) was first treated with epichlorohydrin (ECH) as a crosslinking agent in an acidic medium. (ii) The crosslinked product (CS-E) was treated with polyethyleneimine (PEI) to increase the number of amine group producing aminated chitosan (CS-I). (iii) Finally, the aminated chitosan was then treated with monochloroacetic acid to produce carboxylated chitosan (CS-MC). Therefore, this study was aimed at evaluating the biomedical properties of the newly modified GIC (CS-MC-GIC), as a novel agent that could potentially combat MDR/PDR oral *Candida*. Moreover, this modified GIC was examined to evaluate the compressive strength (CS), diametral tensile strength (DTS), flexural strength (FS), and Vickers microhardness (HV) as a new leading structure in the pharmaceutical field.

## Materials and methods 

### Isolation of *Candida* from oral cavity

In this study, a total of 291 swabs of dental caries were kindly provided by the Clinical Dental Laboratories at Tanta University Hospitals. The clinicians in dental units followed the guidelines and the standard protocols that are compatible with the requirements of the Helsinki Declaration. The provided swabs were transferred to the Lab of Microbiology, Faculty of Science, Tanta University, Egypt, for isolation of *Candida*. The isolates were inoculated onto plates containing selective medium (CHROMagar *Candida*; CAC, CHROMagar Co., Paris, France) and incubated at 37°C for 24–48 hrs. All isolated *Candida* were sub-cultured on Sabouraud dextrose agar (SDA; Ifco Laboratories, Detroit, MI, USA) containing chloramphenicol (300 µg/mL) to avoid bacterial growth, then tested biochemically by germ tube production, API *Candida* system (bioMérieux Vitek, Hazelwood, MO, USA) and their identification was confirmed using VITEK2 automated systems (BioMérieux-Marcy-L´E´toile, France) [51].

### Susceptibility test for *C. albicans* isolates

Antifungal susceptibility testing for isolates of *C. albicans* was performed by the disc diffusion method according to Bauer [52]. For inoculum preparation, each strain was cultured on plates of SDA, and incubated overnight. Then, they were suspended in sterile normal saline solution. The fungal concentration was adjusted to 1 × 10^6^ cells/mL. The prepared fungal inocula were swabbed on the top surface of SDA and then allowed to dry for 10 min. The antifungal discs (Table 1) were placed aseptically over the inoculated agar plates and allowed to diffuse for 15 min at room temperature. All the plates were then incubated for 24 hrs. at 37°C. The zone of growth inhibition (mm) surrounding the discs was measured in order to assess the sensitivity. The findings were interpreted in accordance with the guidelines of the Clinical and Laboratory Standards Institute [53].

**Table 1.** Antifungal resistance pattern of oral *C. albicans* isolates (*n* = 105) from dental caries.

*C. albicans* isolates percentage (%)		Drug resistance Patterns (DRPs)	Type of sensitivity	Strain code
47.5%	32%	AMB, ITC, CLT, MIZ, FLC, MCFG, NYT	MDR	C1,C4,C7,C8,C11,C17,C18,C26,C27,C31,C32,C33,C34,C35,C36,C37,C38,C39,C40,C41,C42,C43,C44,C45,C46,C47,C48,C49,C50,C51,C52,C53,C54,C55,C56,C57,C58, C59, C60,C61, C91,C92,C93,C94,C95,C96,C97,C98,C99,C100
	10%	CLT, MIZ, FLC, MCFG		
	5.5%	ITC, CLT, MIZ, FLC, TRB		
5%		AMB, ITC, CLT, MIZ, FLC, MCFG, NYT, TRB,CSP, FC	PDR	C10, C14, C16, C5, C25
47.5%		ND	S	C2,C3, C6, C9, C12, C13, C15, C19, C20, C21,C22, C23, C24,C28,C29,C30,C62,C63,C64,C65,C66,C67,C68,C69,C70,C71,C72,C73,C74,C75,C76,C77,C78,C79,C80,C81,C82, C83,C84,C85,C86,C87,C88,C89,C90,C101,C102,C103,C104,C105

ND, not detected; AMB, amphotericin; FLC, fluconazole; ITC, itraconazole; CLT, clotrimazole; MIZ, miconazole; MCFG, micafungin; NYT, nystatin; TRB, terbinafine; CSP, caspofungin; FC, flucytosine; MDR was defined as resistance to at least 3 or more antifungal categories; PDR was defined as resistance to all antifungal categories; S, sensitive to antifungals.

### Estimation of some virulence factors of *C. albicans* resistant isolates

#### Biofilm formation

The ability of resistant isolates of *C. albicans* (MDR and PDR) to form biofilm was tested using a colorimetric XTT [2,3-bis (2-methoxy-4-nitro-5sulfophenyl)-2 H-tetrazolium-5-carboxanilide sodium salt] reduction assay [54]. Cell suspension of each strain was prepared in Sabouraud dextrose broth (SDB) at a density of 1 × 10^6^ cells/mL, after that 100 μL was added to each well in micro-titer plates. The plates were incubated at 37°C for 48 h. After incubation, medium was removed from the wells, and non-adherent cells were removed by washing the biofilms three times with sterile phosphate buffered saline (PBS). The wells residual PBS was removed. One hundred microliters of fresh broth, 90 μL of XTT salt solution (0.5 mg/mL) and 10 μL menadione solution (1 mM) were added to each well of prewashed biofilms and incubated in the dark at 37°C for 5 hrs. Biofilm metabolism converts XTT tetrazolium salt to XTT formazan during incubation. The absorbance was then measured spectrophotometrically at 490 nm to determine which strain produced the most biofilm.

#### Neuraminidase enzyme

##### Culture conditions for induction of neuraminidase enzyme

Neuraminidase production medium was prepared as follows (in %): (NH_4_)2HPO_4_ 0.2, NaCl 0.3, KH_2_PO_4_ 0.1, MgSO_4_·7 H_2_O 0.01, FeSO_4_·7 H_2_O 0.002, yeast extract 0.05 and sialic acid (N-acetylneuraminic acid, NANA) 0.5 [55]. Three milliliters of cell suspension (1 × 10^6^ cells/mL) of the most resistant and biofilm producers of *C. albicans* were inoculated separately in 250 mL Erlenmeyer conical flasks containing 100 mL of neuraminidase production medium, which was then shaken at 200 rpm for 6 days at 37°C. After that, neuraminidase enzyme activity was determined daily in a cell-free supernatant as crude enzyme extract.

##### Neuraminidase assay

Neuraminidase activity was determined using the thiobarbituric acid method [56]. Highest neuraminidase production was determined daily for 6 days titrimetrically by mucin hydrolysis for selected isolates to illustrate the optimum incubation period for enzyme production. The assay was standardized as follows: 0.5 mL of crude enzyme, 100 µL of 0.1 M sodium acetate buffer, pH 5.5, and 0.75 mg of mucin as a substrate for neuraminidase enzyme then incubated for 15 min at 37°C. Samples after incubation were treated in a water bath for 30 min at 37°C with 250 µL of the periodate reagent. With 200 µL of the sodium arsenite, the excess of periodate is then reduced. Within 1–2 min, the yellow color of the released iodine has disappeared. Then 2 mL of the reagent thiobarbituric acid (TBA) is applied, and the test sample is heated and covered for 7–5 min in a boiling water bath. In ice-water, the colored solutions are then shaken and cooled with 5 mL of butanol. By a rapid short centrifugation, the two phases were easily separated, and the absorbance was measured at 549 nm. One unit of the enzyme activity was determined as the amount of the enzyme needed to produce 1 µmoL/min of N-acetylneuraminic Acid (NANA) per min under assay condition.

##### Molecular identification

The genomic DNA of selected isolates was isolated using a yeast genomic DNA isolation kit (TakaRa, Japan) according to the manufacturer’s instructions. For PCR amplification, the primers NL1/NL4 and ITS1/ITS4 were used [57]. The amplification products were sequenced by Sangon Biotech (Shanghai, China). The most virulent selected strain SYN-01 shared sequence homology with the closest related yeast strains, according to a BLAST search. MEGA 7.0 software was used for the tree construction.

##### Preparation of experimental groups of modified glass ionomer cements with carboxylated chitosan (CS-MC-GICs)

Five experimental groups were prepared, one pure GIC group was set as (GIC) and four CS-MC-GIC groups, where carboxylated chitosan was added to GIC powder at 2.5%, 5%, 7.5% and 10% (w/w), set as CS-MC-GIC-1, CS-MC-GIC-2, CS-MC-GIC-3 and CS-MC-GIC-4 groups, respectively. The two powders (carboxylated chitosan and GIC) were then completely mixed with DMSO (1%) instead of GIC liquid to create a homogeneous mixture with the same ratio (powder/liquid ratio 3.6:1) as indicated by the manufacturer (GC corporation, Tokyo, Japan). The concentrations were chosen as mentioned previously [50].

##### Anti-candidal activity of CS-MC-GICs groups and determination of cell viability

An agar well diffusion method was performed to determine the activity of experimental groups against the selected strain as described by El-Zawawy et. al [58]. Briefly, the SDA plates were inoculated with 100 μL of the selected strain (1 × 10^6^ cells/mL); with wells of size 8 mm filled with 50 μL of each experimental group. Untreated cells and DMSO (1%) were used as negative and vehicle controls, respectively. Then, these plates were incubated at 37°C for 24 hrs. After the incubation period, the diameter of inhibition zones was measured.

To determine cell viability, the number of viable cells of each experimental group compared to controls was determined using Cell Counting Kit-8 based on monosodium salt WST-8 according to the manufacturer’s protocol (Sigma-Aldrich) [59]. For the assay, 100 µl of cell suspension (1 × 10^6^ cells/mL) were used. Cellular dehydrogenases reduced WST-8 to an orange formazan product that was soluble in the buffer. After 3 hrs., the absorbance of the cell suspension was measured at 450 nm using a TECAN Infinite 200 microplate reader. The amount of formazan produced was directly proportional to the number of living cells.

##### Antibiofilm activity of CS-MC-GICs

The anti-biofilm potential of each experimental group was tested against the selected strain, according to Ramage [60] and minor modifications by El-Zawawy [61]. The selected strain’s cell suspension (1 × 10^6^ cells/mL) was prepared in SDB and added to microtiter plates (100 μL per well) with 100 μL of different CS-MC-GICs groups. As negative and vehicle controls, 100 μL of SDB and 100 μL of DMSO (1%) were added to wells, respectively. Microtiter plates were incubated for 48 hrs. at 37°C and biofilm metabolic activity was determined as mentioned above by colorimetric XTT assay.

##### Neuraminidase inhibitory activity

The neuraminidase inhibitory activity (NIA) was determined by neuraminidase assay as described in section 2.3.2 using 100 μL of serial dilution of each experimental group with different concentrations of mucin (1, 3, 4, 5 and 10 mg/mL) as a substrate. NIA was calculated according to El Zawawy et al. [62]. By plotting the reciprocal of enzyme activity along the y-axis and the reciprocal of substrate concentration along the x-axis, the Lineweaver-Burk plots were created, and the enzyme kinetics was analysed [63]. The trend of lines provides insight into the mechanism of inhibition, whether it is competitive or not.

##### Cytotoxicity of CS-MC-GICs

The cytotoxicity of each experimental group on monkey kidney epithelial cells (Vero; ATCC CCL-81) was evaluated by MTT assay [64] with minor modifications according to Mahmoud and Aly [65] at the Scientific Research Centre and Measurement. Briefly, Vero cells were seeded in 96 well plates of micro-culture (100 μL/well) and incubated for 24 hrs. for adaptation. Cells were permitted to adhere overnight. To test the cytotoxicity of the experimental groups, cells were treated with each group (100 µL/well) compared to untreated cells, DMSO (1%) and DMSO (10%) as negative, vehicle and positive controls, respectively. MTT colorimetric assay determined cell viability by adding 15 µL of MTT/well (final concentration of 500 µg/mL) and incubating for 3 hrs. Then, optical densities (OD) were measured at 570 nm.

#### Testing of mechanical properties of CS-MC-GICs

##### Compressive strength (COS) and diametral tensile strength (DTS)

After mixing each experimental group powder with the liquid at the ratio of (P/L = 3.6 g/1 g) as recommended by the manufacturer, 10 cylindrical specimens for COS testing and 10 for DTS testing were prepared in Teflon molds with the dimensions of 4 mm in diameter and 6 mm in height. The mixed cement was packed, within 60 s after the end of mixing, to the molds with a slight overfills and then gently compressed between two glass plates covered with transparent polystyrene matrix films. One hour after the end of mixing, specimens were removed from the molds, abraded with 800 grit silicon carbide papers to remove the excess material and stored in distilled water at 37°C in an incubator for 24 hrs. Just before testing, specimens of each group were slightly dried with a sheet of damp filter paper (Whatman No. 1). Specimens of both COS and DTS were loaded until fracture at a constant cross-head speed of 1 mm/min [66] using a universal testing machine (Zwick/Roell Z020, Leominster, UK). Prior to examination, each specimen was checked for accurate dimensions using a digital caliper (Mitutoyo, Tokyo, Japan). For COS calculations, the maximum load used to fracture the specimen was divided by the cross-section area to obtain the value of the COS for each specimen in MPa as indicated in ISO 9917:2007 [67] according to the following formula:

$$CS\;=\;P/\pi r^{2}$$

where *P* (N) is the load at fracture and *r* (in mm) the radius of the cylindrical specimen.

The diametral tensile strength was determined from the relationship [67]:

DTS = 2P/πdt

where *P* (N) is the load at fracture, *d* is the diameter and *t* is the thickness (in mm) of the cylindrical specimen.

##### Flexural strength (FS)

A rectangular Teflon mold with dimensions of 2 mm width by 2 mm depth by 25 mm length was used to prepare bar-shaped specimens for flexural strength examination. A total of 10 specimens were prepared from each experimental group. The mixed GIC was packed inside the molds and then covered with a glass microscope slide with a slight hand pressure. After setting, specimens were removed and abraded with 800 grit silicon carbide paper to remove the excess material. All the specimens prepared were stored in distilled water at 37°C in an incubator for 24 hrs. Using a three-point bending method with a 20-mm span and a cross-head speed of 1 mm/min [45], flexural strength was measured by mounting the test assembly on a universal testing machine (Zwick/Roell Z020, Leominster, UK) as outlined in ISO 4049 specification (1999). Prior to examination, the thickness of each specimen was measured using a digital caliper (Mitutoyo, Tokyo, Japan) at the center and at each end of the specimen, and the median thickness was used for cross-sectional area calculations. The load at fracture and the specimen dimensions were used to calculate the flexural strength (**σ**_**f**_) in MPa, according to the following formula [68]:

$$FS\;=\;3PL=2bd^{2}$$

where *P* (N) is the load at fracture, *L* is the distance between the supports, *b* is the width, and *d* is the height of the specimen, all in mm.

##### Vickers microhardness (HV)

For microhardness measurement, 10 disc-shaped specimens (8 mm diameter × 2 mm thickness) were prepared in Teflon molds. Glass microscope slides, covered with transparent polystyrene matrix films, were positioned on the top and bottom surfaces of the specimen and pressed with slight hand pressure to extrude excess material. After complete setting, excess material around the mold was removed by hand-grinding with 800 grit silicon carbide paper. After removal from the mold, specimens were stored in distilled water for 24 h before testing. A Digital Microhardness Tester (Zwick/Roell, IDENTEC, ZHVμ-S, West Midlands, England) was used for hardness measurement by applying a load of 2.9 N force for 15 s [45,47]. All results were generated and reported in HV units using a microhardness tester. The Vickers hardness number (VHN) (kgf/mm^2^) for each specimen was measured according to the following equation [66]:

$$VHN\;=\;1.8544\;\times \;L\;/d^{2}$$

where *L* is the applied load (kgf) and *d* is the mean diagonal length (mm).

Each specimen was subjected to five indentations equally-spaced over a circle. Care was taken to make the indentation not closer than 1 mm to the adjacent indentations or the margin of the specimen. The average of the five indentations per specimen was then calculated, and the mean of 10 specimens was taken as the micro-hardness of the group.

#### Mode of action of CS-MC-GIC-4 (the most effective group)

##### Assessment effect of CS-MC-GIC-4 on Sodium, Potassium and Calcium ions leakage

Ion leakage of sodium, potassium, and calcium was determined, according to Torey [69]. One milliliter of cell suspension of selected strain (1 × 10^6^ cells/mL) was added to 5 mL of CS-MC-GIC-4. After centrifugation at 7000 rpm, the supernatant solution was analysed for the presence of selected ions using a flame photometer at 589 nm, 769 nm and 423 nm, respectively. Untreated cells and DMSO (1%) were used as negative and vehicle controls, respectively.

##### Morphology imaging using scanning electron microscopy (SEM)

Untreated and treated cells with CS-MC-GIC-4 (10 µL) for the selected strain were washed with phosphate buffer solution and air-dried in desiccators [70,71]. The samples were observed under a scanning electron microscope after being coated with gold or palladium (40–60%) (JEOL, JSM–5200 LV, Tokyo, Japan).

##### Statistical analysis

An IBM compatible personal computers with the SPSS statistical package version 20 (SPSS Inc. Released 2011. Armnok, NY: IBM Corp.) was used in data analysis. A one-way analysis of variance (ANOVA) with the significance level established at (*p* ≤ 0.05) was applied for analyzing the results of the studied properties. As significant differences were found between groups of flexural strength and microhardness, Levene’s test for homogeneity of variances was carried out for the data of each property (*p* ≤ 0.05) to choose the appropriate test for multiple comparisons. Equal variances were confirmed (*p* > 0.05); therefore, the Bonferroni test was applied. For comparison between groups, t-test was applied using GraphPad prism (V.8.0.2) software was used. *p ≤ 0.05, **p ≤ 0.01, ***p ≤ 0.001, ****p ≤ 0.0001, While *p* > 0.05 is non-significant (ns).

## Results

### Profile of oral *Candida* species from dental caries

**Figure 2.**
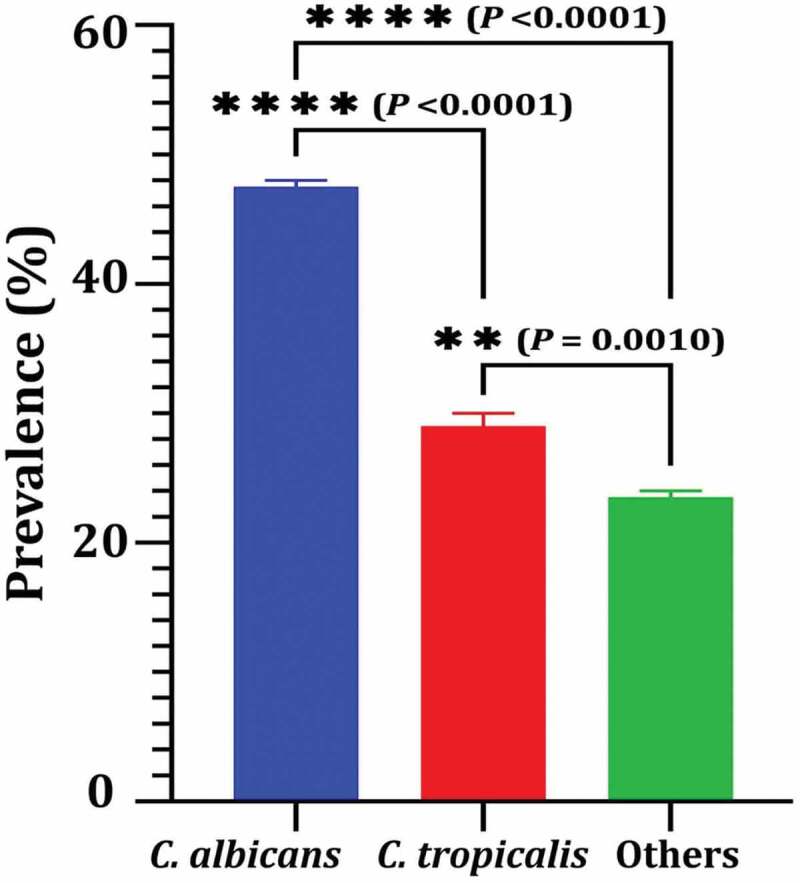
Prevalence of *C. albicans* among other *Candida* species isolated from swab samples of dental caries. Variations between groups were analysed using unpaired and paired t-tests according to the type of obtained data at * *P*≤0.05, ** *P*≤0.01, *** *P*≤0.001, and **** *P*≤0.0001, while *P* > 0.05 is non-significant (ns).

Among 220 oral samples of dental caries, *C. albicans* was found in 47.5% of the samples (Figure 2). *C. albicans* was the most commonly detected species (*n* = 105) followed by *C. tropicalis* (29%), and others (*C. krusei*, *C. dubliniensis* and *C. auris*) detected in 23.5% of the samples.

### Susceptibility of *C. albicans* isolates

Drug resistance patterns (DRPs) of *C. albicans* isolates are shown in Table 1. Fifty isolates of *C. albicans* (47.5%) were proven to be multi-drug resistant (MDR) as they recorded resistance to at least one antifungal in three or more antifungal classes; 5% of *C. albicans* isolates were found to be pan-drug resistant (PDR) as they recorded resistance to all antifungal classes, while, 47.5% of *C. albicans* isolates were sensitive to the used antifungals.

### Virulence factors of resistant *C. albicans* isolates

#### Biofilm activity

**Figure 3.**
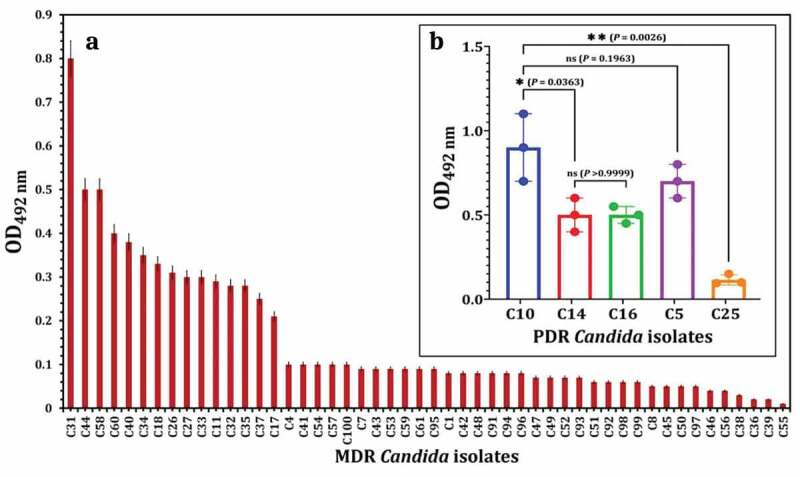
Virulence factors of resistant *C. albicans* isolates. Biofilm activity of MDR *C. albicans* (a) and PDR *C. albicans* (b) in order to select the most potent biofilm-producing *C. albicans* strain. Variations between groups were analysed using unpaired and paired t-tests according to the type of obtained data at * *P*≤0.05, ** *P*≤0.01, *** *P*≤0.001, and **** *P*≤0.0001, while *P* > 0.05 is non-significant (ns).

Biofilm activity was measured for fifty-five resistant *C. albicans* isolates (MDR = 50 isolates and PDR = 5 isolates). Figure 3a shows that 15 isolates of MDR *C. albicans* are the most biofilm producers. Also, four isolates of PDR *C. albicans* are the strongest biofilm producers, as shown in Figure 3b and especially C10 showed the highest biofilm activity among all resistant isolates.

#### Neuraminidase activity

**Figure 4.**
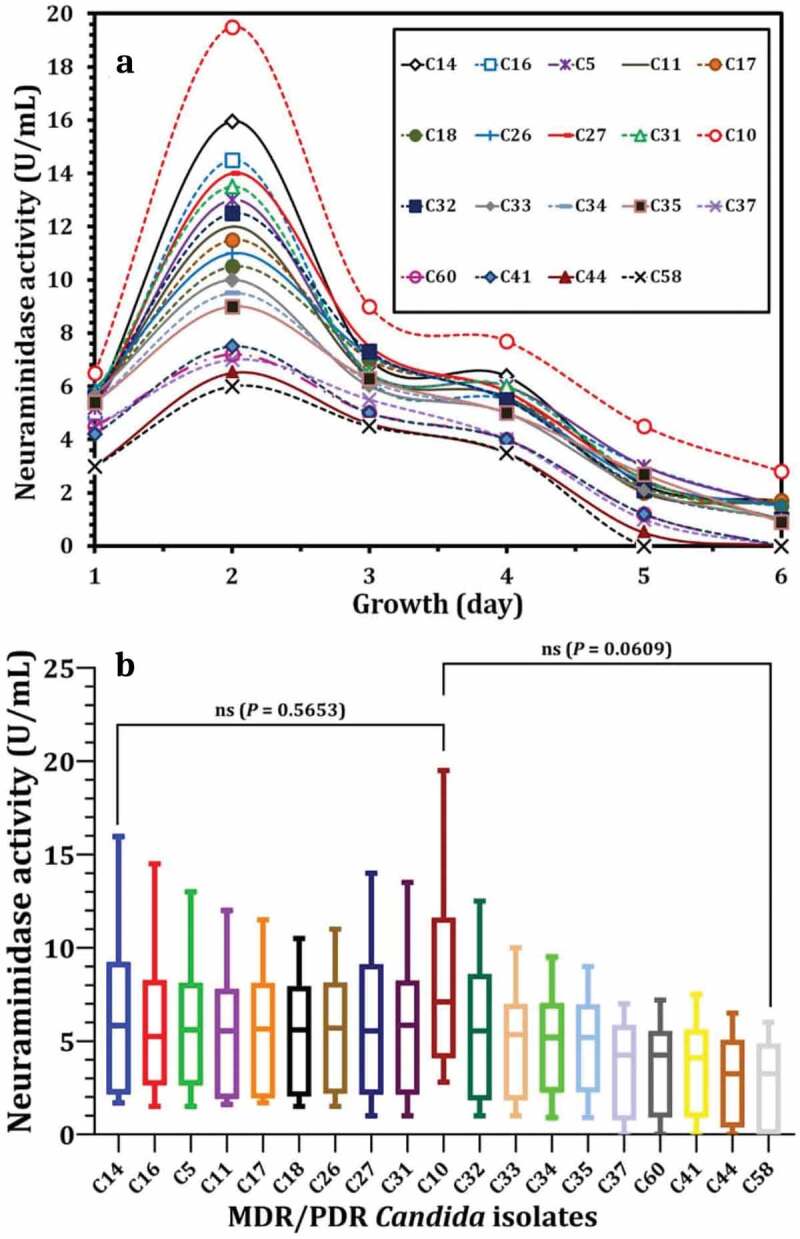
Activity of Neuraminidase produced by MDR/PDR *C. albicans* isolates during their growth for six days (a) and statistical analysis (b) to show the variation in neuraminidase production. Variations between groups were analysed using unpaired and paired t-tests according to the type of obtained data at * *P*≤0.05, ** *P*≤0.01, *** *P*≤0.001, and **** *P*≤0.0001, while *P* > 0.05 is non-significant (ns).

During a period of 6 days, the production of the neuraminidase enzyme was studied in the most resistant and biofilm-producing *C. albicans* isolates (*n* = 19, MDR = 15, PDR = 4). On the second day of growth for all strains, the highest significant production rate of neuraminidase was detected, ranging from 19.5 to 7 U/ml (Figure 4a). As shown in Figure 4b, C10 showed the highest activity rate of neuraminidase.

#### Phylogenetic analysis of selected strain

**Figure 5.**
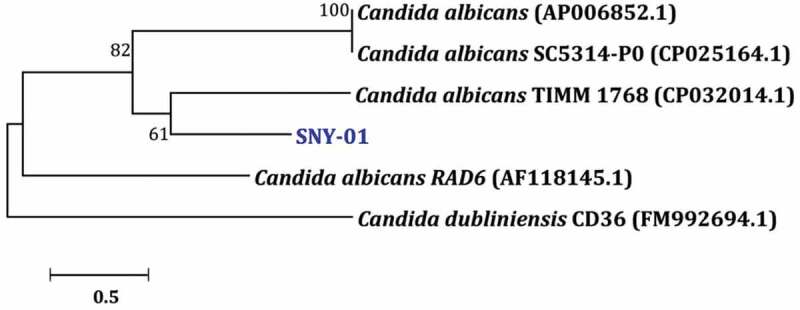
Phylogenetic tree based on 18S rRNA gene sequences inferred using the Neighbor- Joining method and showing the position of *Candida albicans* SYN-01 within closely related taxa. The percentage of replicate trees in which the associated taxa clustered together in the bootstrap test (1000 replicates) are shown next to the branches. The scale bars indicate substitutions per nucleotide position.

The evolutionary history of the selected strain C10 was inferred using the Neighbor-Joining method (Figure 5). This strain stand for molecularly identified species stands for molecularly identified species *Candida albicans* based on GeneBank BLAST comparisons to their closest phylogenetic relatives. Strain SYN-01 (MW856836) showed 98.66% identity to *Candida albicans* strain TIMM 1768 (CP032014), while it showed identity 95.94% identity to *Candida albicans* SC5314 (CP017629) and *Candida albicans* (AP006852). The comparisons to the closest relatives also revealed that the strain *Candida albicans* SYN-01 showed 96.23% identity to *Candida albicans* RAD6 (AF118145). C10 is denoted as Pan-drug resistant *Candida albicans* Strain SYN-01 (PDR-CA-SYN-01).

#### Anticandidal, cell viability and antibiofilm activity of CS-MC-GICs groups

One pure GIC group and four CS-MC-GICs groups were formulated. The anticandidal activity of these five groups has been tested *in vitro* against the most virulent PDR- CA-SYN-01. CS-MC-GICs groups showed significant inhibitory activity against the selected strain compared to GIC group. These groups exhibited distinct differences in susceptibility in a dose-dependent manner. As can be seen in Table 2, the anticandidal activity showed a systematic increase with greater percentage of added CS-MC to GIC in group 4 (CS-MC-GIC-4) against a selected strain. Moreover, CS-MC-GIC-4 showed a significant decrease in cell viability percentage (15%) compared to untreated cells and DMSO (1%) as different controls. Similarly, CS-MC-GICs groups exhibited anti-biofilm activity against selected strain by inhibiting biofilm formation (Table 2). The biofilm formation by the selected strain was reduced significantly with the increase of CS-MC concentration in different groups as CS-MC-GIC-4 showed maximum inhibitory effects on biofilm formation.

**Table 2.** Anticandidal, cell viability and antibiofilm activities of CS-MC-GICs groups.

	Anti-candidal activity	Cell viability	Antibiofilm activity	
CS-MC-GICs experimental groups	Zone of inhibition (mm)	(%)	A _492 nm_	Producing Category
CS-MC-GIC-1	20.0 ± 0.00	60	0.4 ± 0.01	+
CS-MC-GIC-2	31.1 ± 0.00	45	0.1 ± 0.02	-
CS-MC-GIC-3	46.6 ± 0.06	25	0.07 ± 0.01	-
CS-MC-GIC-4	60.8 ± 0.1	15	0.05 ± 0.00	-
GIC	5.0 ± 0.0	96	0.8 ± 0.01	+++
Untreated group	0.0 ± 0.0	100	0.89 ± 0.01	+++
DMSO (1%)	0.0 ± 0.0	100	0.9 ± 0.01	+++
ANOVA $\frac{Fvalue}{Pvalue}$	8152 <0.0001 ****			

**CS-MC-GIC-1**: 2.5% CS-MC, **CS-MC-GIC-2**: 5% CS-MC, **CS-MC-GIC-3**: 7.5% CS-MC, **CS-MC-GIC-4**: 10% CS-MC. **GIC**: GIC group without CS-MC. **Producing category**, -: not a producer, +: weak producer, ++: moderate producer, +++: strong biofilm producer.

#### CS-MC-GIC as a neuraminidase inhibitor

**Figure 6.**
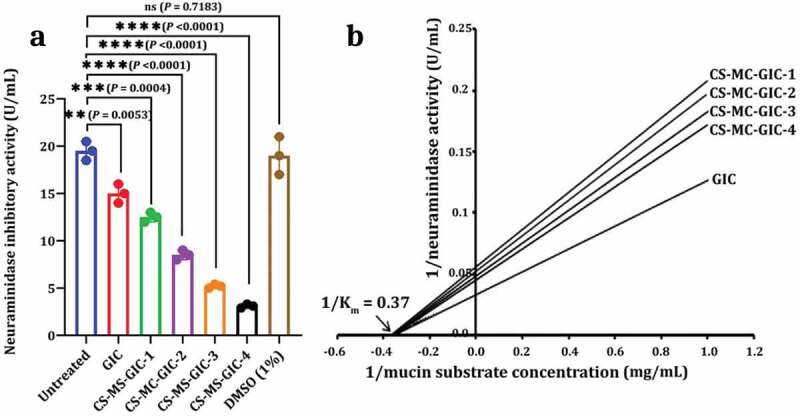
Neuraminidase inhibitor. Inhibitory activity of CS-MC-GICs against neuraminidase activity of PDR *Candida albicans* SYN-01 (PDR-CA-SNY-01) (a) and Lineweaver-Burk plot mechanism of this inhibitor compound (b). Variations between groups were analysed using unpaired and paired t-tests according to the type of obtained data at * *P*≤0.05, ** *P*≤0.01, *** *P*≤0.001, and **** *P*≤0.0001, while *P* > 0.05 is non-significant (ns).

In order to explore the potential of CS-MC-GIC as anti-neuraminidase candidate against PDR-CA-SYN-01, Figure 6a shows a decrease in the neuraminidase activity of the selected strain by increasing the concentration of CS-MC in the experimental groups, as neuraminidase activity decreased from 19.5 U/mL to 3.1 U/mL. According to these results, a Lineweaver-Burk plot was used to detect the mode of inhibition action of CS-MC-GICs on neuraminidase enzyme for selected strains. Figure 6b shows that CS-MC-GIC was a reversible non-competitive neuraminidase inhibitor for selected strains, where the set of lines were found to intersect each other on the x- axis depicting the K_m_ values as km (Michaelis constant) = 2.7 mg/mL and the maximum velocity (V max) decreased by decreasing CS-MC concentration in experimental groups.

#### Cytotoxicity of CS-MC

**Figure 7.**
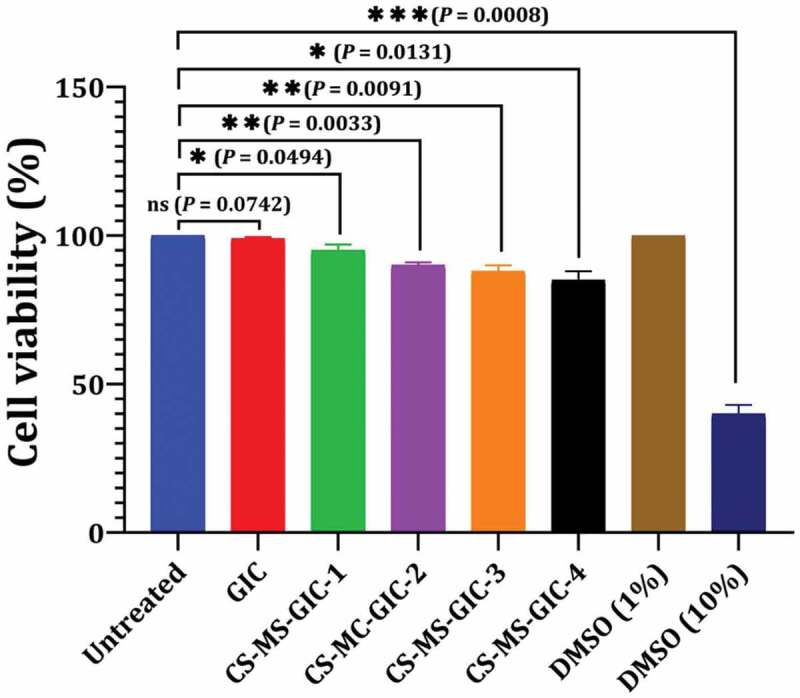
Cytotoxicity of CS-MC-GICs represented as cell viability percentage. Variations between groups were analysed using unpaired and paired t-tests according to the type of obtained data at * *P*≤0.05, ** *P*≤0.01, *** *P*≤0.001, and **** *P*≤0.0001, while *P* > 0.05 is non-significant (ns).

Figure 7 shows lack of cytotoxicity of CS-MC-GICs groups as the percentage viability of Vero cells at the highest treated concentration in CS-MC-GIC-4 was observed to be 90% compared to untreated cells, DMSO (1%) and DMSO (10%) as different controls. This percentage of cell viability of CS-MC-GICs did not show significant toxicity in Vero cells.

#### Investigated mechanical properties

The mean and standard deviations of compressive strength (COS), diametral tensile strength (DTS), flexural strength (FS), and Vickers microhardness (HV) of C-GIC and CS-MC-GICs are listed in Table 3. Results of multiple comparisons (Bonferroni test) are shown by superscript letters, (*p* ≤ 0.05). Mean values of COS ranged between (207 ± 13.38) and (219 ± 12.39) MPa, DTS between (21.11 ± 2.48) and (25.34 ± 2.87) MPa, FS between (24.31 ± 3.11) and (29.54 ± 3.44) MPa and HV between (76.81 ± 5.64) and (83.83 ± 5.17) VHN. Both COS and HV results recorded a systematic reduction of mean values upon increasing the percentage of added chitosan derivative. This reduction was significant in the case of HV but not in COS. The DTS results showed an inconsistent pattern. Group 4, which has a 10% addition of CS-MC, showed a lower mean value than other groups in COS and HV, while FS and DTS results demonstrated a systematic significant increase in mean values upon increasing the added percentage of CS-MC in group 4.

**Table 3.** Mean and standard deviations (in parentheses) of compressive strength, diametral tensile strength, flexural strength and Vickers microhardness of CS-MC-GICs groups.

Properties CS-MC-GICs	Compressive Strength (MPa)	Diametral Tensile Strength (GPa)	Flexural Strength (Mpa)	Vickers Microhardness (VHN)
CS-MC-GIC-1	217 (14.82)^a^	21.35 (3.11) ^a^	25.87 (5.32)^a^	83.25 (7.14) ^a^
CS-MC-GIC-2	213 (10.47) ^a^	19.09 (4.67)^a^	26.14 (7.22)^a,b^	81.91 (7.18)^a,b^
CS-MC-GIC-3	209 (11.35)^a^	23.21 (1.45) ^a^	28.29 (5.18)^a,b^	78.72 (6.21)^a,b^
CS-MC-GIC-4	207 (13.38) ^a^	25.34 (2.87) ^a^	29.54 (3.44)^b^	76.81 (5.64)^b^
GIC	219 (12.39) ^a^	21.11 (2.48)^a^	24.31 (3.11)^a^	83.83 (5.17)^a^

Each value represents the mean of ten specimens. Different superscript letters indicate statistically significant differences between groups of each column (p ≤ 0.05). CS-MC-GIC-1: 2.5% CS-MC, CS-MC-GIC-2: 5% CS-MC, CS-MC-GIC-3: 7.5% CS-MC, CS-MC-GIC-4: 10% CS-MC. GIC: GIC group without CS-MC.

#### Leakage of ions and Morphological changes of PDR-CA-SYN-01 after CS-MC-GIC-4 treatment as a proposed mechanism

**Figure 8.**
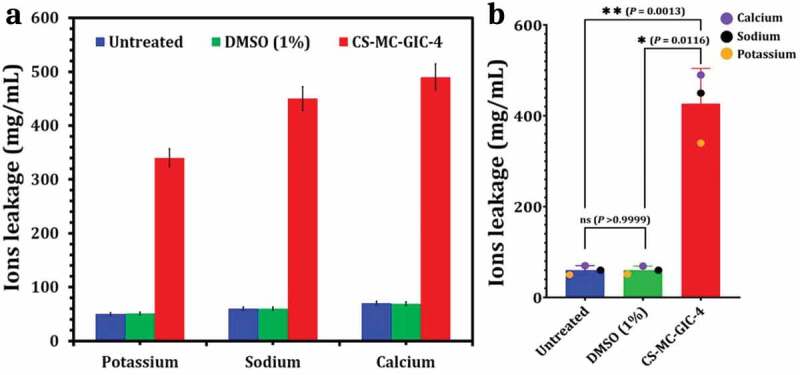
Leakage of ions of PDR-CA-SNY-01 treated with CS-MC-GIC-4 (a) and statistical analysis variation among them (b). Variations between groups were analysed using unpaired and paired t-tests according to the type of obtained data at * *P*≤0.05, ** *P*≤0.01, *** *P*≤0.001, and **** *P*≤0.0001, while *P* > 0.05 is non-significant (ns).

**Figure 9.**
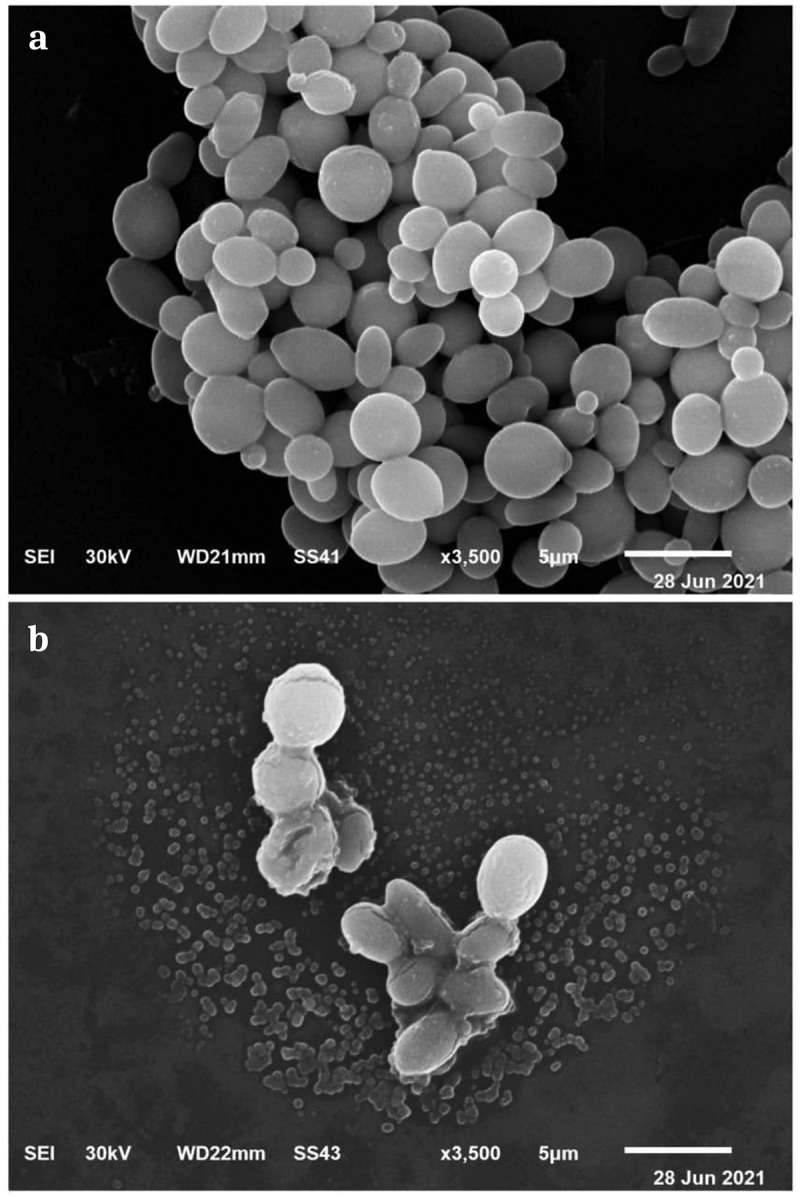
Scanning electron micrograph showing mode of action of CS-MC-GIC-4 against PDR *Candida albicans* SYN-01. Control cells (a) and treated cells (b).

Investigating the possible mode of action of the most effective group (CS-MC-GIC-4) against the PDR-CA-SYN-01 strain, the resulting patterns of potassium, sodium, and calcium ion leakage were estimated and are presented in Figure 8a and b. It was found that CS-MC-GIC-4 was effective in leakage of sodium, potassium, and calcium ions in a selected strain by releasing 390, 480 and 520 mg/mL, respectively. By contrast, untreated CA-SYN-01 and 1% DMSO, which served as negative and vehicle controls, respectively, had small traces of all the selected ions in the culture supernatant. Moreover, significant morphological changes in selected strain were observed when compared with control cells. In control cells (Figure 9a), many oval cells with a smooth appearance were shown, as well as some cells were observed at a budding stage. After CS-MC-GIC-4 treatment, cells appeared as shrunken cells, and eventually completely collapsed cells that were observed (Figure 9b). At this stage, the damage to the cells was beyond repair, and the cells lost their metabolic functions completely.

## Discussion

*C. albicans* was found more frequently on occlusal tooth surfaces than on buccal mucosa or the tongue, owing to its greater affinity for carious tooth structure [72]. In our study, *C. albicans* was isolated from 47.5% of the oral samples. Our findings are similar to those showing a potential role for *C. albicans* in dental caries [73]. This yeast can easily adhere to human dental hard tissues and form biofilms. This yeast also produces acid by metabolizing dietary sugars and carbohydrates, which dissolves hydroxyapatite crystals in the enamel and dentin [74,75]. *Candida* also produces acid at pH levels lower than 4.0 and has a high tolerance to acid [76]. Cariogenicity is determined by several factors, including *Candida’s* ability to adhere to or colonize the tooth surface [77]. *Candida* adherence to oral surfaces is thought to be one of the most important virulence factors, as saliva induces *Candida* germ tube formation [11]. Adherence contributes to candidal persistence in a variety of ways [74]. *Candida* can establish itself within buccal mucosal tissue, bind to, and colonize teeth through the secretion of several extracellular enzymes that promote the invasion of these microorganisms into host cells through the degradation of host mucin in saliva, resulting in mucous membrane inflammation in the buccal cavity [78].

The global emergence of MDR/PDR *Candida* is increasingly limiting the effectiveness of current antifungals and significantly causing treatment failure [79]. Our results showed that among the *C. albicans* isolates from dental caries, one isolate is PDR, which was analysed with ITS2 sequencing and identified as CA-SYN-01. This isolate was suggested to be the most resistant and virulent strain, which secretes neuraminidase with high activity through invading the mucosal surface of the oral cavity, which may establish microbial growth within mucosal epithelial cells, forming a biofilm. Thus, our novel explanation of successful colonization of *Candida* promoting development of dental caries is neuraminidase enzyme which hydrolyzed sialic acid (SA) which is an important structural component of saliva [80]. Sialic acid (SA) is a general term for an acetylated derivative of neuraminic acid that is one of the terminal residues in many glycoproteins and glycolipids [81]. SA protects molecules and cells from protease and glycosidase attack, extending their lifetime and function [82]. The hydrolysis of SA by the neuraminidase enzyme increases cytokines, which causes inflammation [83,84], and also helps *Candida* in the formation of surface-attached biofilm communities as a survival strategy that may result in drug resistance [85,86]. This may be the first study on the production of PDR-CA-SYN-01 for neuraminidase enzyme as an effective tool of dental caries, to penetrate the mucosal surface of the buccal cavity, colonize teeth, and destroy proteinized tissue by the action of extracellular neuraminidase. Consequently, due to the increase in resistance to antifungals, there is an urgent need to improve GIC’s antimicrobial properties against such resistance agents.

Different approaches have been tried to develop restorative materials with enhanced antimicrobial properties. Glass ionomer cements (GICs) are one of those dental materials that have been investigated to improve their defending characteristics. Inhibition of microbial growth around these materials, both *in-vivo* and *in-vitro*, was thought to be due to their capabilities of release of fluoride ions [87]. However, there are different points of view about the effect of fluoride on secondary caries. Despite the presence of some research that has demonstrated that this problem was reduced with fluoride-containing restorations [88,89], others could not prove the inhibiting effect of fluoride after setting. Moreover, other investigations [90,91] concluded that no relation between fluoride release and microbial inhibition was evident and microbial cells may affect the dentin/restoration interface by microleakage causing secondary caries. The improvement of GICs is very valuable both for dental patients and dental clinicians. Therefore, our study proposes a new strategy for preventing dental caries by using CS-MC-GIC. The present study demonstrated that the CS-MC-GICs groups had high anticandidal activities against PDR-CA-SYN-01. The potential antimicrobial action of CS-MC-GIC may be due to the structure of this modified derivative as it has different hydrophilic functional groups, such as hydroxyl, amino, and carboxyl groups. Similarly, in our previous study methyl acrylate chitosan bearing *p*-nitrobenzaldehyde exhibited antibacterial activity due to the hydrogen bond deformation and a substitution of the *p* - nitrobenzaldehyde on the nitrogen atoms [48].

In a trial to explain the ability of CS-MC-GICs to control dental caries, we observed that CS-MC-GICs have strong anti-neuraminidase activity, so this will diminish the ability of the selected strain to invade the dental/oral tissues. Similarly, Rajasekaran et al. [92] reported that *Mussaenda elmeri, Santiria apiculata* and *Anisophyllea disticha* inhibited viral neuraminidase enzymatic activity as there is not any study on inhibition of *Candida* neuraminidase enzyme. Our study also determined that CS-MC-GIC acted as a reversible non-competitive inhibitor for neuraminidase enzyme in PDR-CA-SYN-01. In accordance with our results, Chintakrindi et al. [93] showed that certain flavones and chalcones inhibited the activity of the viral neuraminidase enzyme in a non-competitive manner. Moreover, Silveria et al. [94] recorded that an alkaloidal extract from the stems of *T. divaricata* inhibited acetylcholinesterase activity in a reversible non-competitive manner. In addition, Kong et al. [95] stated that grape seed extract inhibited both α-amylase and α-glucosidase enzymes in a reversible competitive manner. To the best of our knowledge, this study might be the first to evaluate the activity of CS-MC-GICs against PDR-CA-SYN-01, biofilm formation, and neuraminidase activity. Thus, a new avenue for further research and development on this newly discovered CS-MC-GIC for translation into therapeutic strategies has been opened.

Cytotoxicity is considered one of the most important detection assays to evaluate the biological safety of a material for biomedical applications. We postulated that CS-MC-GIC lacked cytotoxicity, which might be due to the presence of amines and carboxyl groups in this modified polymer, which showed good biocompatibility [96,97]. Finally, this work was considered an initial step for searching materials with antimicrobial potential for future use in various dental procedures, as CS-MC-GIC can act as an effective antimicrobial filler and biological activator. Moreover, CS-MC-GICs slightly increased the DTS and decreased the COS. The FS was significantly increased, and the HV showed a slight reduction. The reduction of HV and COS of modified GICs can be attributed to the polymeric nature of the added CS-MC which is lower than the ceramic glass powder of the GIC in terms of mechanical behavior. The slight enhancement of DTS and FS might be explained on the basis that the added polymeric component to GIC reduced the brittleness of the material, which led to enhanced FS and DTS.

The membrane disruption mechanism observed in our study as a possible mechanism of anticandidal activity of CS-MC-GIC-4 against selected strain may be due to the electrostatic interaction between the negatively charged microbial cell surface and the positively charged NH_2_ groups of the modified chitosan. These interactions may compromise membrane permeability and interfere with the synthesis of membrane proteins, thereby affecting the structure and function of the microbial cell [98]. This was similar to observations from other studies with plant lectins [99,100]. Moreover, damaging of microbial membranes and disrupting their functions has been reported by Kenway and Mahmoud [101]. Our results of membrane disruption were confirmed with potassium, sodium and calcium leakage as osmotic balance can be easily affected by the changes in these ions resulting in cell lysis. These ions play a crucial role in activating essential enzymes that function as organic catalysts mediating prominent biochemical reactions [102]. Similarly, Cox et al. [103] studied the influence of tea tree oil on *C. albicans* and drew the conclusion that leakage of potassium ions was an indication of cell membrane structure damage. According to Nagarsekar et al. [104], disruption of the cytoplasmic membrane can be evidenced by leakage of sodium and potassium ions. Hence, the results of CS-MC-GIC may pave the way to confirm the mechanism of action of this novel candidate that can be used in biomedical applications in dental materials.

## Conclusions

Dental caries is a highly prevalent disease all over the world, and the role of *Candida* in dental caries is not yet clear. In this study, 47.5% out of 220 oral samples of dental caries were *C. albicans*. Of these isolates, 47.5% were classified as MDR *C. albicans*, while 5% were PDR isolates. PDR *Candida albicans* SYN-01 showed the highest biofilm and neuraminidase activities among all *Candida albicans* isolates. CS-MC-GICs, a reversible non-competitive neuraminidase inhibitor, showed significant anticandidal and antibiofilm activities against PDR *Candida albicans* SYN-01, along with novel anti-neuraminidase activity. The cytotoxicity assay of CS-MC-GICs confirmed the safety of the modified glass ionomer cements with carboxylated chitosan (CS-MC-GICs) as compared with the control. Overall, the findings of this study showed the potential of CS-MC-GIC-4 as a novel biomaterial candidate with improved biological properties. The study also provides future directions to further understand the mechanism of action of CS-MC-GICs against *Candida albicans* of dental caries at molecular and genetic levels, which may lead to the development of oral therapeutic tools and dental replacement materials against pathogenic *Candida albicans*.
